# The Impact of the G6PD Gene Mutations in Patients with Chronic Hepatitis C Infection Treated with Direct-Acting Antivirals: A Multicenter Observational Study

**DOI:** 10.3390/genes15091116

**Published:** 2024-08-24

**Authors:** Carlo Smirne, Maria Grazia Crobu, Chiara Gerevini, Alessandro Maria Berton, Rachele Rapetti, Barbara Pasini, Paolo Ravanini, Mario Pirisi

**Affiliations:** 1 Internal Medicine Unit, Maggiore della Carità Hospital, 28100 Novara, Italy; chiaragerevini.03@gmail.com (C.G.); rachele.rapetti@med.uniupo.it (R.R.); mario.pirisi@med.uniupo.it (M.P.); 2 Department of Translational Medicine, University of Piemonte Orientale, 28100 Novara, Italy; 3 Laboratory of Molecular Virology, Maggiore della Carità Hospital, 28100 Novara, Italy; mcrobu@cittadellasalute.to.it (M.G.C.); paolo.ravanini@gmail.com (P.R.); 4 Clinical Biochemistry Laboratory, City of Health and Science University Hospital, 10126 Turin, Italy; 5 Division of Endocrinology, Diabetes and Metabolism, City of Health and Science University Hospital, 10126 Turin, Italy; alessandromaria.berton@unito.it; 6 Department of Medical Sciences, University of Turin, 10126 Turin, Italy; barbara.pasini@unito.it; 7 Division of Medical Genetics, City of Health and Science University Hospital, 10126 Turin, Italy

**Keywords:** glucose-6-phosphate dehydrogenase deficiency, favism, anemia, hemolytic, hepatitis C virus, direct-acting antivirals agents, pegylated interferon, ribavirin, drug-related side effects and adverse reactions, liver cirrhosis, sofosbuvir

## Abstract

Following the advent of direct-acting antivirals (DAAs), the treatment of hepatitis C virus (HCV) infection is now rarely challenging. However, data are still limited concerning DAA use in patients affected by glucose-6-phosphate dehydrogenase deficiency (G6PDd). Based on these considerations, the goal of this study was to evaluate the effectiveness and safety of DAAs in this subpopulation. A retrospective multicenter observational study (2015–2023) was conducted on all 2754 consecutive HCV-positive patients treated with first- and second-generation all-oral DAAs, and with a G6PDd diagnosis confirmed by quantitative testing (n = 38). At the treating clinician’s discretion, an enhanced clinical and laboratory follow-up was performed, generally on a monthly basis both during treatment and up to six months after the end of it. Concerning hematochemical parameters, no significant differences were found between any considered time point. In all cases, no treatment-related adverse events were reported, and virologic response rates were as expected without G6PDd. In conclusion, in a large experience which, to the best of our knowledge, is unprecedented in the literature, the treatment of HCV hepatitis with nearly all available DAAs in patients with G6PDd as a comorbidity—a common occurrence in countries such as Italy—proved to be highly effective and safe.

## 1. Introduction

The treatment of hepatitis C virus (HCV) infection is now rarely a challenge because direct-acting antivirals (DAAs) are safe and effective in most patients [1,2,3,4]. This is all the more relevant considering that there is no effective HCV vaccine yet, and reinfection continues to remain a possibility even in developed countries. However, data are still very limited concerning DAA use in patients affected by glucose-6-phosphate dehydrogenase (G6PD) deficiency (d), also known as favism, (formally *hemolytic anemia, G6PD-deficient*: OMIM #300908), an X-linked genetic disorder caused by mutations in the *G6PD* gene which can result in acute hemolytic anemia in the presence of increased reactive oxygen species production. According to the estimates of the World Health Organization (WHO), 7.5% of the world population are carriers of G6PDd and 2.9% are G6PD-deficient. Although most variants have only slightly subnormal red blood cell survival, the Mediterranean variant—observed in Africa, Southern Europe, and several Middle Eastern countries—renders the cells highly susceptible to oxidative stress [5,6,7,8,9,10]. Historically, previous treatment of hepatitis C with standard or pegylated (PEG) interferon (IFN) was generally considered safe in G6PDd patients, although most studies were centered on combination therapies [which included ribavirin (RBV), in turn a well-known factor for dose-dependent intravascular hemolysis] [11,12,13]. Concerning current DAA regimens, while patients with comorbid G6PDd may have been included in registrative trials, specific information on the efficacy and safety in these patients is not available. To the best of our knowledge, the current literature cites only two genotype 4 children treated with ledipasvir/sofosbuvir for 12 weeks, in which no treatment-related serious adverse events were reported [14]. Based on these considerations, the goal of this study was to evaluate the effectiveness and safety of HCV treatments in this special subpopulation.

## 2. Materials and Methods

A retrospective multicenter observational real-life study (from June 2015 to December 2023) was conducted in the liver clinics of six Italian hospitals on all consecutive HCV mono-infected patients treated with first- and second-generation all-oral DAAs, and with a diagnosis of G6PDd in medical history. All subjects gave written informed consent to their participation in the research, which was conducted in strict adherence to the principles of the Declaration of Helsinki of 1975, as revised in 2000. The study protocol was approved by the local institutional Ethics Committee: Comitato Etico Interaziendale Novara, https://comitatoetico.maggioreosp.novara.it/, IRB code CE34/17 113.889 (accessed on 17 July 2024). In more detail, the inclusion criteria were (a) positivity for HCV-RNA; (b) patient age ≥ 18 years and written informed consent to participation in the study; (c) treatment with at least one all-oral DAA regimen; (d) available medical records containing thorough clinical and laboratory information; (e) confirmation of G6PDd with a standardized laboratory method.

Concerning the last point, in all cases, the disease was verified with a quantitative testing for G6PD activity (kinetic assay for the determination of glucose-6-phosphate dehydrogenase activity in erythrocytes, code BCS180955; Sentinel Diagnostics, Milan, Italy), using the Roche COBAS Integra 800 platform (Roche Diagnostics AG, Rotkreuz, Switzerland) [15,16,17,18,19]. The claimed sensitivity and specificity of the test are 97.8 and 97.9%, respectively. The determinations were conducted before any antiviral treatment, and after checking that there were no current or recent hemolytic episodes and/or potential trigger medicines, in order to minimize the risk of false positive results. Following the manufacturer’s instructions, G6PD enzyme activity was determined by measuring the increase in absorbance at 340 nm due to the reduction in NADP^+^, and then expressed as the ratio of enzymatic activity over hemoglobin (Hb) concentration measured in the same sample (IU/gHb). To correctly classify each sample as “normal”, “intermediate”, or “deficient”, the obtained results were compared with the cutoffs reported in the product data sheet, as detailed in Table S1. Briefly, values indicating deficiency in the homozygous adult population for our laboratory were <2.71 IU/gHb in males and <2.95 IU/gHb in females, while results up to 10.22 IU/gHb identified heterozygosity in females. IU/gHb results were also transformed into percentages of relative enzymatic activity, as reported in Table S2.

The test used for serum HCV-RNA detection was Alinity m HCV, which is an in vitro pangenotypic reverse transcription–polymerase chain reaction (RT-PCR) assay for both the detection and quantitation of HCV-RNA from HCV antibody-positive individuals, with a claimed sensitivity of 12 IU/mL and a limit of detection (LoD) of 8.50 IU/mL (Abbott Laboratories, Abbott Park, IL, USA). Patients who did not have a liver elastography (Fibroscan^®^) performed within six months of blood collection were recalled for testing before starting DAA regimens.

For what concerns statistical analysis, continuous variables were expressed as medians and interquartile ranges, and categorical variables as percentages. The Mann–Whitney, Wilcoxon, and Kruskal–Wallis tests were used to compare continuous non-parametric variables, as appropriate. Pearson’s chi-squared test was used to determine whether there was a significant difference between the expected and the observed frequencies in one or more categories. A *p* value of < 0.05 was considered to be significant. All analyses were performed using Stata 18 statistical software (StataCorp LLC, College Station, TX, USA).

## 3. Results

For this study, 2754 HCV-positive subjects were screened. Forty-two Caucasian subjects with a medical history of G6PDd were first identified. After testing for G6PD enzyme activity, four subjects were excluded because they were not confirmed as “deficient”; precisely, two males had a non-classifiable phenotype, one female had a heterozygous phenotype, and one female displayed a normal phenotype. So, ultimately, 38 patients with chronic hepatitis C were selected for this research. To note, a couple of subjects were treated with two consecutive different lines of all-oral DAAs because of a first treatment failure [i.e., they were administered a rescue therapy with sofosbuvir/velpatasvir/voxilaprevir after a previous relapse to sofosbuvir/velpatasvir (n = 1) or glecaprevir/pibrentasvir (n = 1) regimens], so that the total number of analyzed pharmacological regimens amounts to 40. Nearly all past and currently available DAAs are represented in our study population, with the different therapy durations according to the respective data sheets available when they were prescribed: ledipasvir/sofosbuvir (n = 5 for 12 weeks and n = 2 for 24 weeks), ombitasvir/paritaprevir/ritonavir plus dasabuvir (n = 4 for 12 weeks); grazoprevir/elbasvir (n = 1 for 12 weeks); sofosbuvir/daclatasvir (n = 2 for 24 weeks); sofosbuvir/velpatasvir (n = 13 for 12 weeks); glecaprevir/pibrentasvir (n = 11 for 8 weeks); sofosbuvir/velpatasvir/voxilaprevir (n = 2 for 12 weeks, as previously reported). The main baseline clinical, demographic, and virological characteristics of the studied population are reported in Table 1 and Table S2. Table 2 provides some additional information on all the DAA treatments analyzed in this study, while Table S3 describes their main mechanisms of action.

At the treating clinician’s discretion, an enhanced clinical and laboratory (including hemoglobin, reticulocyte, and indirect bilirubin) follow-up was performed, generally on a monthly basis both during treatment and up to six months after the end of it (Table 3). For what concerns hematochemical parameters, no statistically significant differences were found between baseline and any considered time point. In all cases, no treatment-related adverse events were reported; sustained virologic response (SVR) was achieved as normally expected in patients without G6PDd (97%, 92.5% when also considering the two failed treatments after the first line of DAAs). One patient, who had a persistent, mild, predominantly unconjugated hyperbilirubinemia preceding the initiation of antiviral treatment, was then diagnosed with a concomitant Gilbert syndrome, as confirmed by the homozygous polymorphism A(TA)_7_TAA in the promoter of the *UDP-glucuronosyltransferase 1A1* gene.

It is worth noting that a significant proportion of the subjects included in the study were standard or PEG IFN-experienced (29% of the total population) and, consequently, had been exposed to weight-based RBV. Although not formally part of the aims of this study, the medical records clearly showed that, in accordance with what has already been widely described in the literature, the extent of hemolysis, and the consequent severe anemia, occurred without any significant differences to the historical controls without G6PDd. The same data also confirmed the known baseline factors associated with anemia occurrence (such as high ribavirin dosing, presence of advanced liver fibrosis/cirrhosis, older age, female gender, low levels of hemoglobin, concomitant renal failure). In all cases, the treating physicians attributed anemia to a major side effect of that combination therapy for chronic hepatitis C, and not to favism itself.

More interestingly, our study also included a few subjects which had previous failed a first-generation protease inhibitor-based triple therapy (i.e., PEG-IFN + RBV plus boceprevir or telaprevir). Again, bearing in mind that this was also not part of the formal analysis of this research—which was centered instead on all-oral DAA regimens—no severe anemia was reported in these individuals by the prescribing clinicians beyond that commonly expected for such treatments, which are notoriously burdened by some major safety issues. This finding, though anecdotal, still deserves attention because, to the best of our knowledge, it was never before formally described in the literature. All these patients finally obtained SVR after a rescue therapy with sofosbuvir/ledipasvir, as detailed in Table 1.

## 4. Discussion

G6PDd, as previously reported, is a heterogeneous X-linked hereditary genetic defect (with over 200 known pathogenic variants) which makes red blood cells highly vulnerable to oxidative injury and, therefore, susceptible to hemolysis [20]. This defect is one of the most common red cells enzymatic disorders, affecting 400 to 500 million people all over the world and causing neonatal hyperbilirubinemia and chronic hemolytic anemia. Although the majority of patients are asymptomatic, the exposure to oxidative stressors—such as certain drugs, some food (e.g., fava beans), or infections—can trigger an acute hemolysis. The most effective management of G6PDd is to prevent hemolysis by avoiding oxidative stresses [21,22].

Chronic hepatitis C displays, in turn, a worldwide endemicity, affecting an estimated 71 million people across the globe, with prevalence rates varying from 0.5% to 2.5% (the latter ones mainly in the Eastern Mediterranean and Europe) [23]. There are no published data on the exact prevalence of G6PDd in HCV-infected subjects, but it is reasonable to estimate that the presence of this defect in the HCV-positive subpopulation may be comparable to the general population; said in other words, it can be assumed that at least 2 million people worldwide have the copresence of both issues.

Concerning HCV-eradicating treatments, the difficulty in G6PDd has always been to administer drugs that do not induce hemolytic crises. Historically, the first HCV-infection therapeutic standard of care began in 1986 in standard IFN, which was then combined with RBV, and, after 2000, with the PEG- IFN plus RBV association. Both these treatments were often complicated by anemia because of the well-known dose-dependent intravascular hemolysis induced by RBV (and, to a lesser extent, also by IFN itself, which may contribute by suppressing the production of erythroid progenitor cells in the bone marrow) [24,25,26,27,28,29,30,31].

Focusing on G6PDd subjects, no studies were conducted on the exclusive effect of IFN monotherapy. Instead, few studies were performed on combination therapies always focusing, however, more on the main cause of anemia, that is, as previously stated, the hemolytic effect of RBV. What can be deduced is that there is quite a body of evidence showing that these treatments were not harmful, in the sense that the rates of anemia found were not significantly different from those expected in the population without this genetic defect. However, it must be said that the results were somewhat controversial, at least for what concerns standard IFN plus RBV, which, to the best of our knowledge, has been tested in one single American study involving 383 patients, of whom 30 (8%) were G6PDd and developed a more severe hemolytic anemia during the antiviral therapy [32]. In contrast, all studies are concordant regarding the association of PEG-IFN plus RBV but, again, the results should be taken with caution because cumulatively only 56 subjects were analyzed. Interestingly, all of this latter research originated from Italy. In a first pilot study conducted on only four subjects, low membrane protein sulfhydrils prior to therapy, but not G6PDd, was predictive of RBV-induced major hemolysis [11]. The results were confirmed in a larger group from Sardinia, an area with high prevalence of both G6PDd and HCV infection [33,34]. More severe anemia and RBV discontinuation or dose adjustments were not demonstrated in the 26 subjects with G6PDd (23% of the study cohort); SVR rates were also comparable between subjects with G6PDd and without G6PDd [12]. A last study, again carried out in Sardinia, reported similar rates of severe anemia and SVR in the G6PDd arm (26 subjects, 38% of the studied population), confirming the possibility of treating without additional serious consequences [13]. A possible explanation of the discrepant results obtained in the first cited study may reside not so much in a putative more harmful effect of standard IFN compared to PEG-IFN, but in the different studied populations: while all the Italian research included subjects mainly with the Mediterranean variant [i.e., NM_000402.4(G6PD):c.653C>T (p.Ser218Phe)], in the American survey most of the population was of Afro-American or Hispanic origin, thus suggesting that patients with other variants of enzyme deficiency may be more susceptible to the hemolytic effects of RBV [32].

From 2011, the HCV-infection therapeutic standard of care changed thanks to the discovery of DAAs, which were first added to the PEG-IFN plus RBV regimens. Boceprevir and telaprevir (both protease inhibitors) were the initial DAA solutions for HCV, being approved by FDA on 13 May and 23 May, respectively [35,36,37,38]. On November 2013, simeprevir (SIM), another protease inhibitor, was then approved by the FDA, but it still needed to be used in combination with PEG-IFN and RBV [39]. The real turning point came short after in December 2013, when sofosbuvir (the first NS5B polymerase inhibitor as well as the progenitor of the so-called second generation of DAAs) was finally approved in combination with RBV (for genotypes 2 and 3) ± SIM (for genotypes 1 and 4)—being the first all-oral regimens, though with still limited SVR rates—or with PEG-IFN plus RBV (for all genotypes) [40]. Within the next two years, almost all the current armamentarium against hepatitis C became available, with the definitive disappearance of the PEG-IFN. The first was ledipasvir (LDV) (from October 2014), which constituted the first combined regimen (with SOF ± RBV) in one tablet for genotypes 1, 4, 5, and 6 [41,42,43]. Then came the combination ombitasvir/paritaprevir/ritonavir ± RBV for the treatment of patients with genotype 1 (in December 2014, with the addition of dasabuvir) and 4 (from July 2015) [44]. Daclatasvir was the next, in combination with SOF ± RBV: it was the first pangenotypic regimen (also from July 2015) [45,46]. It was followed by the combination of elbasvir and grazoprevir ± RBV, first for patients with genotype 4 and then 1 (from January 2016); this started the third DAA generation, characterized by both high potency and a genetic barrier to resistance. It was then the turn of the combination of SOF/velpatasvir (VEL) ± RBV for adult patients of all genotypes (from July 2016). From here on, it is a more recent history. A rescue therapy for those who failed a previous DAA therapy, i.e., the combination sofosbuvir/velpatasvir/voxilaprevir, became available from July 2017. Finally, the pangenotypic combination glecaprevir/pibrentasvir was approved in August 2017. No new drugs have been licensed since then [47,48,49,50]. Table 2 provides some additional information on all these past and current treatments, pointing out those that were analyzed in the present study for what concerns the subgroup of subjects with G6PDd. As can be seen, all were analyzed, except for asunaprevir (which, however, has never been available in Western Europe) and SIM. With regard to the first-generation protease inhibitors (boceprevir and telaprevir), the evidence derived is only indirect, since this study was focused on all-oral treatments, as discussed above.

What is evident from the literature is that DAAs have proven to be not only effective but also largely safe drugs, especially since PEG-IFN and RBV could be discontinued (thus referring to the second wave of the second generation and the entire last generation of DAAs), including those subjects with relevant comorbidities [3,51,52,53] or advanced liver disease/cirrhosis [54,55,56]. However, there are some recent preliminary data, mostly on SOF ± RBV, suggesting that—at least in animal models—DAAs may also cause deleterious effects on various metabolic pathways involved in the physiologic function of mitochondria, the endocrine system, or immune responses [57,58,59]. In any case, no relevant hematological side effects have ever been reported in humans and, in particular, the problem of anemia—so frequent in the pre-DAA era—has now completely disappeared. But when focusing on individuals with G6PDd, it appears that this specific issue has never been addressed. As a matter of fact, a search of the published medical literature was conducted regarding use of DAAs in patients with comorbid G6PDd (also generically investigating for possible interactions with drugs likely to cause hemolysis in patients with favism), and no real pertinent information was identified. As described above, only one single abstract presented in 2018 at the Asian Pacific Association for the Study of the Liver (APASL) mentioned the use of a SOF-based therapy (LDV/SOF) in just two Egyptian teens with G6PDd, without further details [14]. Moreover, none of the DAA datasheets ever contained any specific mention or warning about favism, and all the pharmaceutical companies that were contacted were also unable to provide any indication in this respect, including post-marketing reports.

When analyzing most phase 3 clinical trials evaluating the safety and efficacy of various DAAs, as previously cited, it appears that patients were generally excluded from participation if they had clinically significant abnormalities other than HCV infection. This—as is standard practice—was always based upon the results of a medical history, physical examination, vital signs, laboratory profile, and electrocardiogram, and possibly made a patient an unsuitable candidate for the specific study in the opinion of the investigator. Moreover, patients with an uncontrolled cardiac, respiratory, gastrointestinal, hematologic, neurologic, psychiatric, or other medical disease or disorder unrelated to the existing HCV infection were also excluded from any trial participation [60,61,62,63,64,65,66]. In conclusion, it is reasonable to state that—while patients with comorbid G6PDd may have been included in some of the clinical trials for DAAs—specific information on the efficacy and safety of DAAs in these subjects was not yet available until now and constituted an unfulfilled scientific gap.

What clearly emerged from our research is that none of the DAAs, neither old- nor new-generation, showed any problem of tolerability in G6PDd patients. Indeed, both their safety profile and efficacy rate were perfectly comparable to those described in the general population of HCV-positive subjects. Specifically, not a single case of hemolysis was found in the study population. Moreover, DAAs were confirmed to be safe and manageable even in the subgroup of cirrhotic patients. So, this real-life study has the merit—although well behind the time of the marketing of these drugs—to confirm what was already known in common clinical practice by most clinicians, namely that no special precautions or enhanced follow-up should be undertaken in subjects with G6PDd when undergoing such treatments. Incidentally, what might seem like a possible limitation of our research, i.e., the fact that only subjects who had a pre-existing diagnosis of favism in their medical history were analyzed, could actually be considered as an advantage. In fact, taking into account what is the normal expected prevalence of G6PDd in the Caucasian population, it can be estimated that, in our whole cohort of HCV-positive subjects (almost 3000 subjects), the individuals affected by the disease were as high as 80, instead of the 38 actually studied [6]. And since in the entire screened population, both the drug-related problems emerging from the analysis of all medical records and the few adverse events reported to the drug regulatory authorities, were never related to anemia/hemolysis issues, it is reasonable to argue that this may actually be an element that further corroborates the safety of these treatments.

If anything, the only point that remains not formally clarified by this study is whether populations with a higher prevalence of African American or Hispanic subjects may have a greater susceptibility to hemolysis, as appeared to be the case for RBV, since our entire case series involved Caucasian Italian subjects, who are therefore likely affected by the Mediterranean-type G6PDd [32]. However, as mentioned earlier, the fact that there has never been any report of adverse events worldwide only confirms that DAAs are safe, regardless of mutations in the *G6PD* gene that may exist within specific ethnic groups.

## 5. Conclusions

In a large multicenter-center experience which, to the best of our knowledge, is unprecedented in the literature, treatment of HCV hepatitis with nearly all available different DAA regimens in patients with G6PDd as a comorbidity—a common occurrence in countries such as Italy—proved to be highly effective and safe.

## Figures and Tables

**Table 1:** Baseline patient characteristics. Total number of recruited patients: 38; total number of considered antiviral treatments: 40. Data are presented as median (range) for continuous variables and as frequency (%) for categorical variables.

	Variable	Local Laboratory NR
Age, years	58 (48–68)	-
Gender, n (M, F)	26 (68), 12 (32)	-
HCV genotype, n (1a, 1b, 2, 3)	4 (11), 11 (29), 15 (39), 8 (21)	-
HCV-RNA, ×10^3^ IU/mL	1818 (972–2554)	negative
Liver elastography, KPa	7.4 (4.8–10.3)	≤5.0
Hepatic cirrhosis, n	8 (21)	-
MELD, score	8 (6–10)	≤6.0
Hemoglobin, g/L	145 (127–151)	115–157
Reticulocytes, %	1.20 (1.00–1.40)	0.50–2.17
G6PD activity (37 °C), IU/gHb	1.38 (0.30–1.84)	-
Males, IU/gHb	1.32 (0.80–1.94)	≥9.52
Females, IU/gHb	1.37 (0.62–1.82)	≥10.22
G6PD activity (37 °C), %	13.86 (8.40–17.99)	100
Males, %	12.95 (7.51–20.37)	100
Females, %	13.44 (6.06–17.60)	100
AST, IU/L	36 (32–53)	0–40
ALT, IU/L	47 (24–81)	0–40
Total bilirubin, mg/dL	1.20 (0.95–2.10)	0.30–1.20
Indirect bilirubin, mg/dL	0.82 (0.45–1.60)	0.30–0.95
Creatinine, mg/dL	0.77 (0.63–0.85)	0.60–1.10
INR, Units	1.02 (0.97–1.02)	0.80–1.20
Previously treated with standard IFN + RBV, n	3 (8)	-
Previously treated with PEG-IFN + RBV, n	8 (21)	-
Administered DAA regimens, n	40 (100)	-
LDV/SOF, n ^1^	7 (18)	-
OMB/PAR/RIT + DAS	4 (10)	-
GRZ/ELB	1 (3)	-
SOF/DCL	2 (5)	-
SOF/VEL	13 (32)	-
GLE/PIB	11 (27)	-
SOF/VEL/VOX ^2^	2 (5)	-
Therapy duration, weeks	12 (12–12)	-
Post-treatment virological outcome ^3^		
SVR, n	37 (97)	-
Relapse, n	1 (3)	-

Abbreviations: alanine transaminase (ALT); aspartate transaminase (AST); boceprevir (BOC); dasabuvir (DAS); daclatasvir (DCL); elbasvir (ELB); glecaprevir (GLE); glucose-6-phosphate dehydrogenase (G6PD); grazoprevir (GRZ); hepatitis C virus (HCV); interferon (IFN); international normalized ratio (INR); international units (IU); ratio of enzymatic activity over hemoglobin concentration measured in the same sample (IU/gHb); ledipasvir (LDV); model for end-stage liver disease (MELD); normal range (NR); ombitasvir (OMB); paritaprevir (PAR); pegylated (PEG); pibrentasvir (PIB); red blood cells (RBCs); ribavirin (RBV); ritonavir (RIT); sofosbuvir (SOF); sustained virological response (SVR); TEL (telaprevir); velpatasvir (VEL); voxilaprevir (VOX). ^1^ in subjects with no response to previous PEG-IFN + BOC + RBV (n = 2) or TEL + BOC + RBV (n = 5). ^2^ in subjects with a relapse to previous SOF/VEL (n = 1) or GLE/PIB (n = 1) regimens. ^3^ not considering the two failures to the first line of DAAs.

**Table 2:** Main current and past DAA regimens for HCV treatment. Molecules were analyzed both individually and in combination therapies. The column on the right shows the treatments that were analyzed in this study on patients with G6PDd; in bold the antiviral treatments currently recommended by most international guidelines are indicated.

Molecule	NS Protein Target	Daily Dosage (mg)	DAA Treatment—Recommended Combination Regimens	Tested in This Study ^1^
**First-generation DAA**				
**Protease inhibitors**				
BOC	NS3	2400	PEG-IFN + RBV + BOC	✔ ^2^
TEL	NS3/4A	2250	PEG-IFN + RBV + TEL	✔ ^2^
**Second-generation DAA**				
**NS5A polymerase inhibitors**				
LDV	NS5A	90	LDV/SOF ± RBV	✔
DCL	NS5A	60	DCL + SOF ± RBV	✔
OMB	NS5A	25	OMB/PAR/RIT + DAS ± RBV	✔
** NS5B polymerase inhibitors**				
DAS	NS5B	500	OMB/PAR/RIT + DAS ± RBV	✔
SOF	NS5B	400	PEG-IFN + RBV + SOF ^3^ SOF + RBV ^3^	
** Protease Inhibitors**				
PAR	NS3/4A	150	OMB/PAR/RIT + DAS ± RBV	✔
SIM	NS3/4A	150	PEG-IFN + RBV + SIM SOF + SIM ± RBV	
BMS-650032 ^4^	NS3	200	BMS-650032 + DCL PEG-IFN + RBV + BMS-650032 + DCL	
**Third-generation DAA**				
**NS5A polymerase inhibitors**				
VEL	NS5A	100	**SOF/VEL ± RBV** **SOF/VEL/VOX**	✔ ✔
PIB	NS5A	120	**GLE/PIB**	✔
ELB	NS5A	50	**EBR/GZR ± RBV**	✔
** Protease Inhibitors**				
GRZ	NS3/4A	100	**EBR/GZR ± RBV**	✔
VOX	NS3/4A	100	**SOF/VEL/VOX**	✔
GLE	NS3/4A	300	**GLE/PIB**	✔

Abbreviations: asunaprevir (BMS-650032); boceprevir (BOC); direct-acting antivirals (DAAs); dasabuvir (DAS); daclatasvir (DCL); elbasvir (ELB); glecaprevir (GLE); glucose-6-phosphate dehydrogenase (G6PD); grazoprevir (GRZ); hepatitis C virus (HCV); ledipasvir (LDV); nonstructural (NS); ombitasvir (OMB); paritaprevir (PAR); pegylated interferon (PEG-IFN); pibrentasvir (PIB); ribavirin (RBV); ritonavir (RIT); simeprevir (SIM); sofosbuvir (SOF); TEL (telaprevir); velpatasvir (VEL); voxilaprevir (VOX). ^1^ only RBV-free treatments were included in this study. ^2^ indirect evidence (not part of the formal protocol analysis of this study). ^3^ current combination therapies with SOF are reported in the “Third generation DAA” section, as they are commonly used in clinical practice. ^4^ commercialized only in Asia and Russia.

**Table 3:** Hemoglobin, reticulocyte, and indirect bilirubin course during antiviral therapy. Data are presented as median (range) for continuous variables and as frequency (%) for categorical variables.

	TW4	TW8 ^1^	TW12 ^2^	ET	FUP4	FUP12	FUP24 ^3^
Hemoglobin, g/L	141 (124–152)	143 (127–151)	140 (122–152)	146 (128–154)	143 (127–151)	141 (126–152)	140 (127–150)
Reticulocyte, %	1.1 (0.9–1.4)	1.0 (1.0–1.5)	1.2 (1.1–1.5)	1.1 (1.0–1.4)	1.2 (1.1–1.3)	1.0 (1.0–1.4)	1.1 (0.9–1.4)
Indirect bilirubin, mg/dL	0.85 (0.49–1.65)	0.90 (0.45–1.61)	0.92 (0.51–1.62)	0.89 (0.49–1.62)	0.87 (0.47–1.59)	0.84 (0.45–1.60)	0.86 (0.44–1.63)

Abbreviations: therapy week 4 (TW4); therapy week 8 (TW8); end of treatment (ET); FUP4, post-treatment follow-up at 4 weeks; FUP12, post-treatment follow-up at 12 weeks; FUP24, post-treatment follow-up at 24 weeks. ^1^ for treatments lasting more than 8 weeks. ^2^ for treatments lasting more than 12 weeks. ^3^ data available for 28/40 treatments.

## Data Availability

All data generated or analyzed during this study are included in this published article.

## Supplementary Materials

The following supporting information can be downloaded at: https://www.mdpi.com/article/10.3390/genes15091116/s1, Table S1: Cutoff values for correct interpretation of the results of IU/gHb; Table S2: Transformation of IU/gHb into percentages of glucose-6-phosphate dehydrogenase enzymatic activity. (a) male subjects. (b) female subjects. Data of column 3 are presented as frequency (%). Table S3: DAA molecular mechanisms of action by HCV target sites.
