# Supplementary material for: The Impact of the G6PD Gene Mutations in Patients with Chronic Hepatitis C Infection Treated with Direct-Acting Antivirals: A Multicenter Observational Study
Source: Genes (Basel). 2024 Aug 24;15(9):1116. doi: 10.3390/genes15091116 (PMC11431558; doi:10.3390/genes15091116)
Supplement: Supplementary file 1 [file genes-15-01116-s001.zip › Table S1.pdf]

Table S1. Cutoff values for correct interpretation of the results of IU/gHb.

Subject category	Minimum value	Maximum value
Normal males	≥ 9.52	-
Deficient males	0.10	2.71
Normal females	≥ 10.22	-
Heterozygous females	3.55	10.22
Deficient females	0.10	2.95

Abbreviation: ratio of glucose-6-phosphate dehydrogenase enzymatic activity over hemoglobin concentration measured in the same sample.
