# Supplementary material for: The Impact of the G6PD Gene Mutations in Patients with Chronic Hepatitis C Infection Treated with Direct-Acting Antivirals: A Multicenter Observational Study
Source: Genes (Basel). 2024 Aug 24;15(9):1116. doi: 10.3390/genes15091116 (PMC11431558; doi:10.3390/genes15091116)
Supplement: Supplementary file 1 [file genes-15-01116-s001.zip › Table S2.pdf]

Table S2. Transformation of IU/gHb into percentages of glucose-6-phosphate dehydrogenase enzymatic activity. (**a**) male subjects. (**b**) female subjects. Data of column 3 are presented as frequency (%).

**Phenotype**		**IU/gHb**	**Affected subjects, n**
	(**a**) **Males**		
Normal	9.520	100.00	-
Not classifiable	9.000	94.54	-
Not classifiable	8.000	84.03	-
Not classifiable	7.000	73.53	-
Not classifiable	6.000	63.03	-
Not classifiable	5.000	52.52	-
Not classifiable	4.000	42.02	-
Not classifiable	2.856	30.00	-
Deficient	2.710	28.47	6 (23)
Deficient	2.000	21.01	7 (27)
Deficient	1.000	10.50	9 (35)
Deficient	0.100	1.05	4 (15)
	(**b**) **Females**		
Normal	10.220	100.00	-
Heterozygote	10.000	97.85	-
Heterozygote	9.000	88.06	-
Heterozygote	8.000	78.28	-
Heterozygote	7.154	70.00	-
Heterozygote	6.000	58.71	-
Heterozygote	5.000	48.92	-
Heterozygote	4.000	39.14	-
Deficient	2.950	28.86	3 (25)
Deficient	2.000	19.57	1 (8)
Deficient	1.000	9.78	5 (42)
Deficient	0.100	0.98	3 (25)

Abbreviation: ratio of glucose-6-phosphate dehydrogenase enzymatic activity over hemoglobin concentration measured in the same sample.
