# Supplementary material for: The Impact of the G6PD Gene Mutations in Patients with Chronic Hepatitis C Infection Treated with Direct-Acting Antivirals: A Multicenter Observational Study
Source: Genes (Basel). 2024 Aug 24;15(9):1116. doi: 10.3390/genes15091116 (PMC11431558; doi:10.3390/genes15091116)
Supplement: Supplementary file 1 [file genes-15-01116-s001.zip › Table S3.pdf]

Table S3. DAA molecular mechanisms of action by HCV target sites [1].

HCV target protein name(s)	HCV target protein biological function(s)
NS3/4A protease	The NS3/4A serine protease is a non-covalent, heterodimer complex formed by two HCV-encoded proteins, the N-terminal serine protease domain of NS3 (catalytic subunit) and the NS4A cofactor (activation subunit). NS3 serine protease is involved in the cleavage of HCV polyprotein precursor, while NS4A protein is a cofactor essential for the activity of the NS3 protease.
NS5A polymerase	It exerts functionally essential effects in the regulation of viral assembly and egress. Moreover, it is critical for viral genome replication, and is thought to interact directly with both NS5B and viral RNA. Finally, it is a key mediator in regulating host cell function and activity upon HCV infection.
NS5B polymerase	It is a RNA-dependent RNA polymerase that plays a critical role in HCV replication. The function of this enzyme is to catalyze the polymerization of ribonucleoside triphosphates (rNTP) during viral RNA replication. There are two main sub-classes of NS5B polymerase inhibitors: (**a**) nucleotide analogues that mimic the natural substrate and induce chain termination when incorporated into the new RNA (e.g., sofosbuvir); (**b**) non-nucleotide inhibitors that bind to the allosteric sites on the enzyme and impair its function (e.g., dasabuvir).

Abbreviations: hepatitis C virus (HCV); nonstructural (NS); ribonucleic acid (RNA); ribonucleoside triphosphates (rNTP).

[1] Three nonstructural (NS) proteins (NS3/4A, NS5A, and NS5B) are important for HCV replication and, therefore, represent important targets for inhibition by DAA. As per their target sites of action, DAA are thus divided in three main classes. The first includes NS3/4A inhibitors, the second NS5A inhibitors, and the last NS5B inhibitors.
